# Chondroid Syringoma of the Nasal Ala: A Case Report

**DOI:** 10.7759/cureus.110223

**Published:** 2026-06-04

**Authors:** Younes Tamim, Yassine Berrada, Taha Yassine Aaboudech, Mariame Meziane, Benzekri Laila

**Affiliations:** 1 Dermatology, University Hospital Center Ibn Sina/Mohammed V University of Rabat, Rabat, MAR; 2 Pathology, University Hospital Center Ibn Sina/Mohammed V University of Rabat, Rabat, MAR

**Keywords:** chondroid syringoma, cutaneous adnexal tumor, dermatopathology, dermoscopy, mixed tumor of the skin

## Abstract

Chondroid syringoma, also known as mixed tumor of the skin, is a rare benign cutaneous adnexal neoplasm characterized by both epithelial and mesenchymal differentiation. It most commonly occurs in the head and neck region and usually presents as a solitary, slow-growing, painless dermal or subcutaneous nodule. Because of its nonspecific clinical and dermoscopic presentation, the diagnosis is rarely suspected before histopathological examination. We report a case of a 73-year-old male with no significant medical history who presented with a progressively enlarging flesh-colored nodule of the nasal ala evolving over one year. Clinical examination revealed a well-demarcated, firm, painless, round nodule measuring 7 mm in diameter, adherent to the overlying skin, with a smooth surface. Dermoscopic examination revealed a central pigmented structureless area, peripheral white dots, linear vessels, and whitish areas, resulting in a nonspecific dermoscopic pattern. The main clinical differential diagnoses included nodular basal cell carcinoma, epidermal inclusion cyst, amelanotic nevus, neurofibroma, and chondroid syringoma. An excisional biopsy was performed. Histopathological examination showed a benign adnexal tumor composed of numerous ductal and tubular epithelial structures embedded within a myxoid to chondromyxoid stroma, consistent with chondroid syringoma. Surgical margins were free of tumor. No recurrence was observed after two years of follow-up. This case highlights the importance of considering chondroid syringoma in the differential diagnosis of long-standing nodular lesions of the nose and emphasizes the role of complete surgical excision and histopathological confirmation.

## Introduction

Chondroid syringoma, also referred to as mixed tumor of the skin, is a rare cutaneous adnexal neoplasm showing both epithelial and mesenchymal differentiation. Its microscopic architecture resembles that of pleomorphic adenoma of the salivary glands, with epithelial structures embedded in a myxoid, chondroid, or chondromyxoid stroma. The term “chondroid syringoma” was introduced by Hirsch and Helwig in 1961 because of the presence of sweat gland elements associated with a cartilage-like stromal component [1].

Clinically, chondroid syringoma usually presents as a solitary, firm, slow-growing, painless dermal or subcutaneous nodule. The lesion is most frequently located in the head and neck region, particularly on the nose, cheek, scalp, upper lip, and chin [2-7]. However, its clinical presentation is nonspecific, and the diagnosis is often difficult to establish before histopathological examination. It may mimic several benign or malignant cutaneous lesions, including basal cell carcinoma, epidermal inclusion cyst, melanocytic nevus, neurofibroma, or other adnexal tumors [2,4,6,7].

We report a case of benign chondroid syringoma located on the nasal ala in a 73-year-old male, successfully treated by complete surgical excision, with no recurrence after two years of follow-up.

## Case presentation

A 73-year-old male with no significant medical history presented with a solitary lesion on the nasal ala that had been evolving for one year. The lesion initially appeared as a small flesh-colored papule measuring approximately 2-3 mm and gradually increased in size, reaching 7 mm at presentation. This corresponded to an estimated average growth rate of approximately 0.3-0.4 mm per month. The patient reported no pain, bleeding, ulceration, or rapid enlargement.

**Figure 1:**
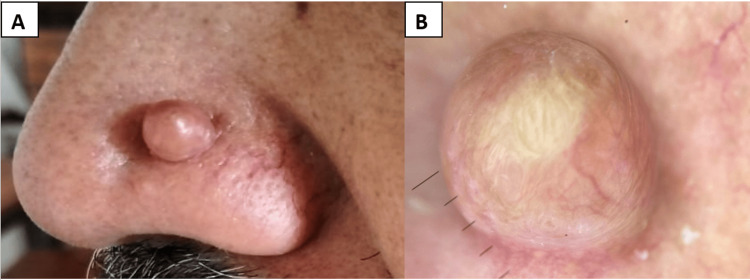
Clinical and dermoscopic features of the nasal ala lesion. (A) Clinical image showing a well-demarcated, flesh-colored, smooth, dome-shaped nodule located on the nasal ala. (B) Dermoscopic examination showing a nonspecific pattern with a central structureless whitish-yellow to pigmented area, peripheral white dots, linear vessels, and whitish areas.

Clinical examination revealed, in a Fitzpatrick skin type IV patient [8], a well-demarcated, round, firm, painless nodule located on the nasal ala. The lesion was adherent to the overlying skin and measured approximately 7 mm in diameter. Its surface was smooth and flesh-colored, without ulceration or pigmentation (Figure 1).

General physical examination showed no palpable lymphadenopathy and no organomegaly. Dermoscopic examination of the nodule revealed a central pigmented structureless area, peripheral white dots, linear vessels, and whitish areas, resulting in a nonspecific dermoscopic pattern (Figure 1).

The clinical differential diagnoses included nodular basal cell carcinoma, epidermal inclusion cyst, amelanotic nevus, neurofibroma, other adnexal tumors, and chondroid syringoma. Nodular basal cell carcinoma was considered because of the patient’s age, the facial location, and the slow enlargement of a firm nodule; however, the absence of ulceration, pearly borders, prominent telangiectasia, and arborizing vessels on dermoscopy made this diagnosis less typical. An epidermal inclusion cyst was considered because the lesion was well circumscribed and firm, but the absence of a central punctum, fluctuation, or previous inflammatory episodes argued against it. An amelanotic nevus was considered due to the flesh-colored appearance, although the progressive enlargement in an elderly patient and the absence of a typical melanocytic dermoscopic pattern made it less likely. Neurofibroma was also considered because it may present as a skin-colored papule or nodule, but the firm consistency, adherence to the overlying skin, and absence of a buttonhole sign did not support this diagnosis. Other adnexal tumors and chondroid syringoma remained possible because of the slow-growing, well-demarcated nodular presentation and nonspecific dermoscopic findings. Therefore, an excisional biopsy was performed for diagnostic confirmation and treatment.

**Figure 2:**
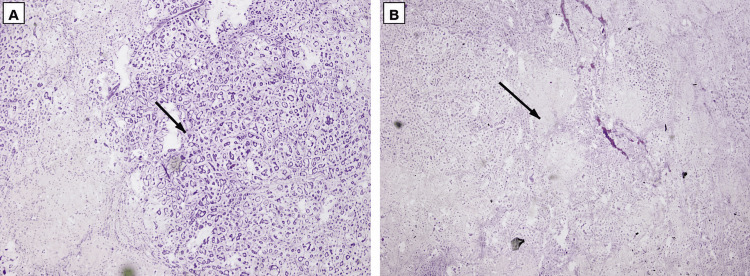
Histopathological features of chondroid syringoma. (A) Hematoxylin and eosin staining showing numerous ductal and tubular epithelial structures within a pale myxoid to chondromyxoid stroma. The black arrow highlights the ductal/tubular epithelial component. (B) Hematoxylin and eosin staining showing a biphasic adnexal tumor with epithelial structures and abundant myxoid/chondromyxoid stroma. The black arrow highlights the stromal component. Original magnifications: A ×100; B ×40.

Histopathological examination revealed a benign adnexal neoplasm composed of numerous ductal and tubular epithelial structures embedded within an abundant myxoid to chondromyxoid stroma, consistent with chondroid syringoma (Figure 2).

Surgical margins were free of tumor. Immunohistochemistry was not performed because the excisional biopsy showed typical morphology, with well-formed ductal/tubular epithelial structures embedded within a myxoid to chondromyxoid stroma and no atypical features. The postoperative course was uneventful, and no recurrence was observed after two years of follow-up.

## Discussion

Chondroid syringoma is an uncommon cutaneous adnexal tumor that shares histopathological similarities with pleomorphic adenoma of the salivary glands, particularly its biphasic epithelial and stromal architecture [1,3]. At the molecular level, some overlap with salivary gland pleomorphic adenoma has been reported, particularly involving PLAG1 rearrangements; however, cutaneous mixed tumors appear to be genetically heterogeneous [9]. Histologically, chondroid syringoma is composed of epithelial elements and a mesenchymal stromal component, which may be myxoid, chondroid, fibrous, or hyaline [1,3]. Molecular testing was not performed in our case because the histopathological findings were typical of benign chondroid syringoma.

Chondroid syringoma usually occurs in adults, most often in middle-aged or older patients, with a male predominance reported in several series, with some series reporting a male-to-female ratio of up to 5:1 [2,3]. A recent systematic review identified 347 cases of cutaneous chondroid syringoma and confirmed the predominance of benign tumors and a predilection for the head and neck region [10]. Nasal and facial involvement is clinically relevant because nodular lesions in this area are more commonly suspected to be basal cell carcinoma, epidermoid cyst, melanocytic lesions, neurofibroma, or other adnexal tumors. Several cases involving the face and nose have been reported, but the diagnosis generally remains histological [4,6,7].

The clinical presentation is typically nonspecific. Chondroid syringoma usually appears as a small, slow-growing, painless, non-ulcerated dermal or subcutaneous nodule. In our patient, the lesion presented as a firm, flesh-colored, painless nodule of the nasal ala, initially measuring approximately 2-3 mm and reaching 7 mm after one year, corresponding to an estimated average growth rate of approximately 0.3-0.4 mm per month. Dermoscopy showed a nonspecific pattern, with a central pigmented structureless area, peripheral white dots, linear vessels, and whitish areas. Dermoscopic data on chondroid syringoma remain limited; in a recent systematic review, reported dermoscopic features were heterogeneous and included vascular structures, white or yellowish-white areas, and structureless areas [10]. The findings in our case were consistent with this nonspecific dermoscopic spectrum and did not allow a definitive clinical diagnosis, supporting the need for histopathological confirmation.

Histopathological examination remains the cornerstone of diagnosis. The classic microscopic features include ductal or tubuloalveolar epithelial structures, nests or cords of epithelial cells, and an abundant myxoid to chondromyxoid stromal matrix [1,3]. Immunohistochemical studies support the biphasic nature of the tumor, with epithelial and myoepithelial differentiation contributing to the characteristic stromal matrix [11].

Several diagnostic pitfalls should be considered, particularly in facial lesions. Chondroid syringoma may mimic basal cell carcinoma, both clinically and histologically [12]. However, basal cell carcinoma typically shows basaloid tumor islands with peripheral palisading and stromal retraction clefts, whereas chondroid syringoma shows true ductal/tubular epithelial differentiation within a myxoid to chondromyxoid stroma. The histological differential diagnosis also includes myoepithelioma, chondroma, and pleomorphic adenoma-like lesions [13]. Myoepithelioma may show myxoid or chondroid stroma but usually lacks well-formed ductal/tubular epithelial structures. Chondroma lacks epithelial differentiation, whereas pleomorphic adenoma is excluded by the absence of a connection with salivary gland tissue. Malignant chondroid syringoma should be suspected in the presence of infiltrative growth, cytological atypia, increased mitotic activity, necrosis, or recurrence.

Immunohistochemistry is not required when the morphology is typical; however, it may be useful when the biphasic architecture is poorly developed, when ductal/tubular differentiation is focal or difficult to identify, or when the biopsy is small or fragmented. It may also help in tumors with prominent myxoid or chondroid stroma and limited epithelial structures, particularly when the differential diagnosis includes cutaneous myoepithelioma, basal cell carcinoma with stromal myxoid change, or other adnexal tumors. In such settings, cytokeratins and epithelial membrane antigen may highlight the epithelial/ductal component, whereas S-100 protein, smooth muscle actin, p63, and calponin may support myoepithelial differentiation [14,15].

The main treatment is complete surgical excision. This approach allows both diagnostic confirmation and definitive treatment. Benign chondroid syringoma generally has an excellent prognosis when completely excised, whereas incomplete excision may be associated with local recurrence [2-4]. Malignant chondroid syringoma is rare. A systematic review identified 51 reported cases, with local recurrence and metastasis described in a substantial proportion of cases [16]. Features suggesting malignant potential include rapid growth, large tumor size, infiltrative margins, cytological atypia, necrosis, frequent mitoses, recurrence, and location outside the head and neck region, particularly on the trunk or extremities [16-18].

In the present case, the small size of the lesion, its slow estimated growth rate, the absence of ulceration, the nasal location, free surgical margins, and the absence of recurrence after two years of follow-up supported the benign nature of the tumor. Complete excision was both diagnostic and curative.

## Conclusions

Chondroid syringoma should be considered in the differential diagnosis of persistent, slow-growing nodules of the head and neck region, especially when clinical and dermoscopic findings are nonspecific. Histopathological examination is essential for diagnosis, and complete surgical excision with clear margins remains the treatment of choice to prevent recurrence.

## References

[1] Hirsch P, Helwig EB (1961). Chondroid syringoma. Mixed tumor of skin, salivary gland type. Arch Dermatol.

[2] Yavuzer R, Başterzi Y, Sari A, Bir F, Sezer C (2003). Chondroid syringoma: a diagnosis more frequent than expected. Dermatol Surg.

[3] Wan H, Xu M, Xia T (2018). Clinical and pathological study on mixed tumors of the skin. Medicine (Baltimore).

[4] Chen AH, Moreano EH, Houston B, Funk GF (1996). Chondroid syringoma of the head and neck: clinical management and literature review. Ear Nose Throat J.

[5] Gotoh S, Ntege EH, Nakasone T, Matayoshi A, Miyamoto S, Shimizu Y, Nakamura H (2022). Mixed tumour of the skin of the lower lip: a case report and review of the literature. Mol Clin Oncol.

[6] AlSaidan L, Sarkhouh M, Alenezi A, AlSabah H, Al Aradi I, Alterki A (2024). Chondroid syringoma of the nose: a rare case report and literature review. Int J Surg Case Rep.

[7] Abil S, Bouhllab J, Zarkik S (2012). Chondroid syringoma of the face. Ann Dermatol Venereol.

[8] Fitzpatrick TB (1988). The validity and practicality of sun-reactive skin types I through VI. Arch Dermatol.

[9] Bahrami A, Dalton JD, Krane JF, Fletcher CD (2012). A subset of cutaneous and soft tissue mixed tumors are genetically linked to their salivary gland counterpart. Genes Chromosomes Cancer.

[10] Di Guardo A, Balampanos CG, Gargano L, Giordano D, Capalbo A, Persechino F, Persechino S (2024). Clinical and dermoscopic characteristics of cutaneous chondroid syringoma: a systematic review. Dermatol Pract Concept.

[11] Dominguez Iglesias F, Fresno Forcelledo F, Soler Sanchez T, Fernandez García L, Herrero Zapatero A (1990). Chondroid syringoma: a histological and immunohistochemical study of 15 cases. Histopathology.

[12] Linares González L, Aguayo Carreras P, Rueda Villafranca B, Navarro-Triviño FJ (2020). Chondroid syringoma mimicking basal cell carcinoma. Actas Dermosifiliogr (Engl Ed).

[13] Chon J, Laub P, Alhalaseh Y, Ogrodnik J (2024). Case of eccrine chondroid syringoma of the upper lip. BMJ Case Rep.

[14] Macagno N, Sohier P, Kervarrec T, Pissaloux D, Jullie ML, Cribier B, Battistella M (2022). Recent advances on immunohistochemistry and molecular biology for the diagnosis of adnexal sweat gland tumors. Cancers (Basel).

[15] Hornick JL, Fletcher CD (2004). Cutaneous myoepithelioma: a clinicopathologic and immunohistochemical study of 14 cases. Hum Pathol.

[16] Zufall AG, Mark EJ, Gru AA (2023). Malignant chondroid syringoma: a systematic review. Skin Health Dis.

[17] Harrist TJ, Aretz TH, Mihm MC Jr, Evans GW, Rodriquez FL (1981). Cutaneous malignant mixed tumor. Arch Dermatol.

[18] Lal K, Morrell TJ, Cunningham M, OʼDonnell P, Levin NA, Cornejo KM (2018). A case of a malignant cutaneous mixed tumor (chondroid syringoma) of the scapula treated with staged margin-controlled excision. Am J Dermatopathol.

